# Prognostic values of inhibitory κB kinases mRNA expression in human gastric cancer

**DOI:** 10.1042/BSR20180617

**Published:** 2019-01-15

**Authors:** David Timothy Gayed, Jayant Wodeyar, Zi-Xiang Wang, Xiang Wei, Yi-Yi Yao, Xiao-Xi Chen, Zhou Du, Ji-Cai Chen

**Affiliations:** 1 School of the First Clinical Medical Sciences, The First Affiliated Hospital of Wenzhou Medical University, Wenzhou Medical University, Wenzhou, China; 2 BMedSci, University of Sydney, Sydney, Australia; 3 Department of General Surgery, The First Affiliated Hospital of Wenzhou Medical University, Wenzhou, China

**Keywords:** Gene Expression Omnibus, gastric cancer, Inhibitory kappa B Kinases, nuclear factor kappa B, prognosis

## Abstract

**Background**: Inhibitory κB kinases (IKKs) play a key role in modulating proinflammatory and growth stimulating signals through their regulation of the nuclear factor κB (NF-κB) cascade. Therefore, the level of expression of IKKs represents a viable prognostic predictor with regard to various pathological processes. The prognostic value of IKKs expression in gastric cancer remains unclear. **Methods**: We used the ‘Kaplan–Meier plotter’ (KM plotter) online database, to explore the predictive prognostic value of individual IKKs members’ mRNA expression to overall survival (OS) in different clinical data including pathological staging, histology, and therapies employed. **Results**: Our results revealed that a higher mRNA expression of inhibitor of NF-κB kinase subunit α (IKKα) was correlated to better OS, whereas higher mRNA expression of IKKβ, inhibitor of NF-κB kinase subunit γ (IKKγ), inhibitor of NF-κB kinase subunit ε (IKKε), and suppressor of IKKε (SIKE) were generally correlated to unfavorable OS in gastric cancer. Increased mRNA expression of IKKε also showed better outcomes in stage IV gastric cancer. Further a correlation between elevated levels of mRNA expression of both IKKε and SIKE was found to have favorable OS in diffuse type gastric cancer. It was also revealed that high expression of SIKE had favorable OS when treated with other adjuvant therapies, while worse OS when treated only with 5FU therapy. **Conclusion**: Our results suggest that mRNA expression of individual IKKs and SIKE are associated with unique prognostic significance and may act as valuable prognostic biomarkers and potential targets for future therapeutic interventions in gastric cancer.

## Introduction

With an estimated 1313000 cases diagnosed in 2015 and 819000 deaths, gastric cancer remains the fifth leading cause of cancer worldwide and the third leading cause of mortality due to cancer [1]. Often asymptomatic in its early stages, it is an insidious disease, with many cases already at an advanced stage at the time of diagnosis [2,3]. The prognosis for such patients is often poor, with the 5-year survival rate estimated to be less than 10% worldwide [1]. Treatment modalities include surgery, chemotherapy, and radiotherapy. More recently, monoclonal antibodies such as trastuzumab and ramucirumab have shown promise when used as an adjuvant with other therapeutic modalities [4]. Nevertheless, there remains a pressing need for new therapeutic agents to improve patient outcomes.

Targetted therapeutics such as trastuzumab owe much of their success to recent advances in the understanding of the mechanisms of carcinogenesis. Commonly used in breast cancer, trastuzumab targets and inhibits human epidermal growth factor receptor 2 (HER2), and gained FDA approval for use in gastric cancer in 2014. Another potential therapeutic target, recently implicated in cancer growth and progression, is the transcription factor NF-κB as well as the pathways that facilitate its activation [5,6].

NF-κB has a long understood physiological role in modulating innate and immune responses to infection [5]. More recent evidence, however, has shone new light on its considerable potential for oncogenesis; it can promote survival, proliferation, and invasiveness in cancer cells by inducing the production of anti-apoptotic proteins such as B-cell lymphoma-2 (Bcl-2) and inhibitor of apoptosis protein (IAP)-1/2, promoters of mitogenesis such as Cyclin D1, and invasive proteases such as matrix metalloproteinase-95–7. Normally retained in an inactive state in the cytosol by inhibitor proteins known as inhibitors of κB (IκB), the activation of NF-κB to the nucleus requires the phosphorylation and consequent enzymatic degradation of IκB by IκB kinase (IKK), following which it undergoes nuclear translocation to exercise its transcriptive functions [6]. This event may be triggered by various stimuli, including cytokines such as tumor necrosis factor (TNF), interleukin-1 (IL-1), as well as bacterial and viral products such as lipopolysaccharide (LPS), such as that produced by *Helicobacter pylori* in the case of gastric cancer [6,7]. Normally controlled by a series of negative feedback loops, NF-κB activity becomes dysregulated in cancer cells. The reasons for this could be linked to mutations leading to marked increases in NF-κB expression and activity, or prolonged exposure to NF-κB-activating stimuli [6,8].

The IKK family comprises three isoforms known as inhibitor of nuclear factor κB (NF-κB) kinase subunit α (IKKα), inhibitor of NF-κB kinase subunit β (IKKβ), and the regulatory subunit, inhibitor of NF-κB kinase subunit γ (IKKγ/NEMO), also known as the classical IKKs, as well as the non-classical IKKs, inhibitor of NF-κB kinase subunit ε (IKKε) and TANK-binding kinase 1 (TBK1) [9]. IKKε and TBK1, while not essential to NF-κB activation, nevertheless have a role to play in the growth and proliferation of many cancers [5,9]. Another protein, SIKE (suppressor of IKKε), performs an anti-inflammatory role by suppressing IKKε and TBK1, effectively preventing the transcription of type 1 interferons [10,11].

Recent studies have examined the role of the various IKK subunits in cancers of the skin, oral cavity, nasopharynx and lung, amongst various others [12]. The role of IKK in gastric cancer, however, has thus far not been studied. To that end, we attempted to investigate the prognostic value of IKKs and SIKE expression in patients with gastric cancer. The results of our investigation could help to improve our understanding of gastric cancer on a molecular level, highlight areas for further study, as well as identification of targets for therapeutic research.

## Materials and methods

The correlation of individual IKKs mRNA expression with overall survival (OS) was assessed using an online Kaplan–Meier plotter (KM plotter) database that was established using gene expression data and survival information from the Gene Expression Omnibus (GEO, including GSE14210, GSE15459, GSE22377, GSE29272, GSE51105, GSE62254). Recently, KM plotter was established with more than 54000 genes that have been validated in gastric cancer, breast cancer, ovarian cancer, lung cancer, and liver cancer. The database provided the clinical data including stage, lauren classification, differentiation degree, gender, perforation, HER2 status, and treatment of gastric cancer patients. In the present study, clinical data, such as stage, lauren classification, differentiation degree, gender, HER2 status, and treatment of gastric cancer patients were collected in this database. We chose best probe set of IKKs to obtain Kaplan–Meier survival plots, and hazard ratio (HR), 95% confidence interval (CI), and log rank *P*-value were determined and displayed on the webpage. A *P*-value <0.05 was considered statistically significant.

## Results

**Figure 1:**
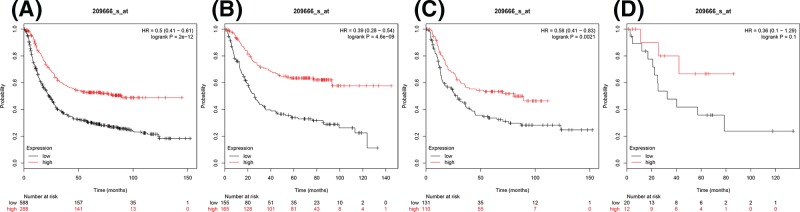
The prognostic value of IKKα expression in gastric cancer (**A**) All the patients; (**B**) intestinal cancer patients; (**C**) diffuse cancer patients; and (**D**) mixed cancer patients.

For investigating survival-associated IKKs in 876 gastric cancer patients in total, four subtypes of IKKs and SIKE were pooled in www.kmplot.com. First, the prognostic value of IKKα was analyzed (Figure 1). For IKKα, Affymetrix ID is 209666_s_at. OS curves were plotted for all gastric cancer patients (*n*=876) (Figure 1A), for intestinal histologic type (intestinal type) (*n*=320) (Figure 1B), diffuse histologic type (diffuse type) (*n*=241) (Figure 1C), and mixed histologic type (mixed type) (*n*=32) (Figure 1D). Elevated expression of *IKKα* mRNA was significantly associated with better OS in all gastric cancer patients, intestinal type, and diffuse type cancer, with HR = 0.5 (0.41–0.61), *P*=2e-12 (Figure 1A), HR = 0.39 (0.28–0.54), *P*=4.6e-09 (Figure 1B), and HR = 0.58 (0.41–0.83), *P*=0.0021 (Figure 1C), respectively. Whereas no significant correlation with OS was shown in mixed type (HR = 0.36, 95% CI 0.1–1.29, *P*=0.1) (Figure 1D).

**Figure 2:**
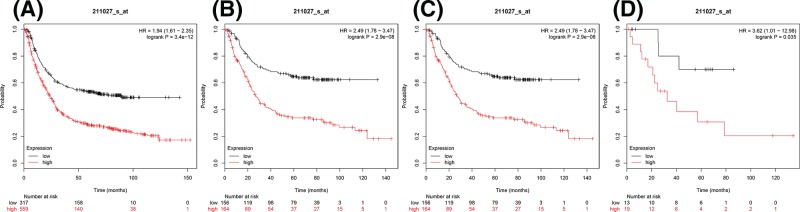
The prognostic value of IKKβ expression in gastric cancer (**A**) All the patients; (**B**) intestinal cancer patients; (**C**) diffuse cancer patients; and (**D**) mixed cancer patients.

Next, the prognostic significance of IKKβ (Affymetrix ID: 211027_s_at) mRNA expression was. *IKKβ* mRNA expression level revealed a significantly correlated and worse OS amongst all gastric cancer patients, HR = 1.94 (1.61–2.35), *P*=3.4e-12 (Figure 2A). The elevated expression of IKKβ also showed significant and unfavorable outcomes in all histological types, intestinal type HR = 2.49 (1.78–3.47), *P*=2.9e-08 (Figure 2B), diffuse type HR = 2.49 (1.78–3.47), *P*=2.9e-08 (Figure 2C), and mixed type HR = 3.62 (1.01–12.98), *P*=0.035 (Figure 2D).

**Figure 3:**
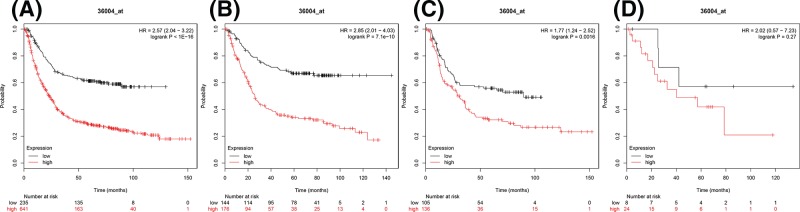
The prognostic value of IKKγ expression in gastric cancer (**A**) All the patients; (**B**) intestinal cancer patients; (**C**) diffuse cancer patients; and (**D**) mixed cancer patients.

The prognostic significance of *IKKγ* mRNA expression (Affymetrix ID: 36004_at.) was also evaluated using the database. Elevated IKKγ expression similarly showed a significant and worse OS within all gastric cancer patients as well as in intestinal type and diffuse type, HR = 2.57 (2.04–3.22), *P*<1E-16 (Figure 3A), HR = 2.85 (2.01–4.03), *P*=7.1e-10 (Figure 3B), and HR = 1.77 (1.24–2.52), *P*=0.0016 (Figure 3C), respectively. However, the difference of IKKγ in mixed type could not distinguish worse OS or better, HR = 2.02 (0.57–7.23), *P*=0.27 (Figure 3D).

**Figure 4:**
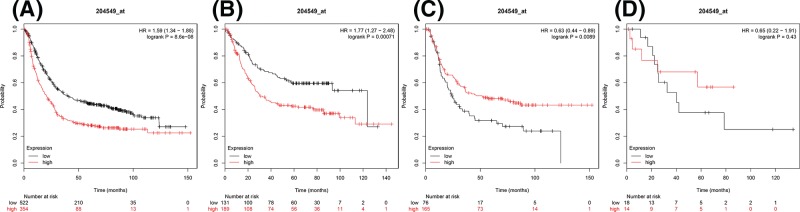
The prognostic value of IKKεexpression in gastric cancer (**A**) All the patients; (**B**) intestinal cancer patients; (**C**) diffuse cancer patients; and (**D**) mixed cancer patients.

Figure 4 demonstrates the prognostic value of IKKε (Affymetrix ID: 204549_at). *IKKε* mRNA expression was again significantly correlated with worse OS for all gastric cancer patients, HR = 1.59 (1.34–1.88), *P*=8.6e-08 (Figure 4A), intestinal type patients likewise showed a worse OS, HR = 1.77 (1.27–2.48), *P*=0.00071 (Figure 4B), however in diffuse type patients the significant difference showed better OS with elevated IKKε expression, HR = 0.63 (0.44–0.89), *P*=0.0089 (Figure 4C). There was no significant correlation in mixed type HR = 6.5 (0.22–1.91), *P*=0.43 (Figure 4D).

**Figure 5:**
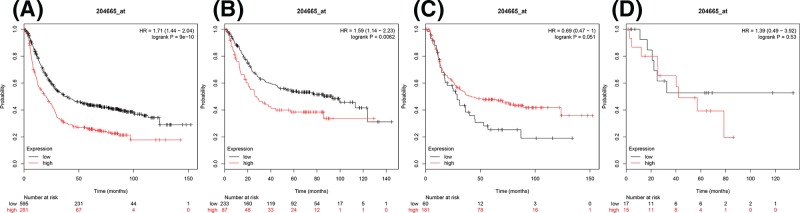
The prognostic value of SIKEexpression in gastric cancer (**A**) All the patients; (**B**) intestinal cancer patients; (**C**) diffuse cancer patients; and (**D**) mixed cancer patients.

Furthermore, the prognostic significance of SIKE was attained through the database (Affymetrix ID: 204665_at). Increased expression of SIKE mRNA was significantly correlated with worse OS for all gastric cancer, HR = 1.71 (1.44–2.04), *P*=9e-10 (Figure 5A) similarly poor OS was noted in intestinal type, HR = 1.59 (1.14–2.23), *P*=0.0062 (Figure 5B). However, elevated expression of SIKE mRNA in diffuse type and mixed type was not significantly correlated with OS, HR = 0.69 (0.47–1), *P*=0.051 (Figure 5C) and HR = 1.39 (0.49–3.92), *P*=0.53 (Figure 5D), respectively.

The correlation of IKKs gene expression level with mortality assessed at 50 months is outlined in Table 1. Only IKKα showed lower mortality (51%), with high expression group, *P*<0.001 when compared with low expression group (73%), whereas all other IKKs subgroups showed higher mortality with high expression group.

**Table 1:** Correlation of IKKs gene expression level with mortality of 50 months

IKKs	Expression	Case	Survival	Mortality	*P*-value
IKKα	Low	588	157	0.732993	<0.001
	High	288	141	0.510417	
IKKβ	Low	317	158	0.501577	<0.001
	High	599	140	0.766277	
IKKγ	Low	235	135	0.425532	<0.001
	High	641	163	0.74571	
IKKε	Low	522	210	0.597701	<0.001
	High	354	88	0.751412	
SIKE	Low	595	231	0.611765	<0.001
	High	281	67	0.761566	

Due to the striking correlation found between IKKs level of expression and OS in gastric cancer, further analysis was undertaken to associate the IKKs subgroups with pathological grade (Table 2), HER2 expression (Table 3), treatment strategy (Table 4), gender (Table 5), cell differentiation (Table 6). As seen in Table 2, IKKα was associated with better OS in pathological grades I through IV. Grade I having the lowest HR = 0.29 (0.11–0.8), *P*=0.011 and grade IV having the highest HR = 0.62 (0.41–0.92), *P*=1.3e-05. In contrast, IKKβ overexpression corresponded to worse OS in all pathological grades. With regard to IKKγ overexpression only stages I, II, and III were significantly correlated and had a worse OS showing HR = 4.6 (1.59–13.27), *P*=0.0019, HR = 3.13 (1.6–6.12), *P*=0.00044 and HR = 2.45 (1.67–3.59), *P*=2.1e-06, respectively. At last, SIKE overexpression only showed a significant correlation in stage III, with HR = 1.69 (1.26–2.26), *P*=0.00037.

**Table 2:** Correlation of IKKs gene expression level with OS in different pathological stages in gastric cancer patients

IKKs	Pathological grade	Cases	HR (95% CI)	*P*-value
IKKα	I	67	0.29 (0.11–0.80)	0.011*
	II	140	0.43 (0.23–0.78)	0.0044*
	III	305	0.46 (0.33–0.66)	1.3e-05*
	IV	148	0.62 (0.41–0.92)	0.018*
IKKβ	I	67	3.7 (1.16–11.75)	0.018*
	II	140	2.62 (1.43–4.79)	0.0012*
	III	305	2.02 (1.45–2.81)	2.3e-05*
	IV	148	1.47 (1.01–2.16)	00.49*
IKKγ	I	67	4.60 (1.59–13.27)	0.0019*
	II	140	3.13 (1.60–6.12)	0.00044*
	III	305	2.45 (1.67–3.59)	2.1e-06*
	IV	148	1.43 (0.97–2.11)	0.071
IKKε	I	67	4.78 (1.35–16.87)	0.0074*
	II	140	1.84 (0.93–3.66)	0.077
	III	305	1.45 (1.07–1.95)	0.015*
	IV	148	0.52 (0.34-0.81)	0.003*
SIKE	I	67	1.62 (0.6–4.39)	0.34
	II	140	1.82 (0.98–3.36)	0.053
	III	305	1.69 (1.26–2.26)	0.00037*
	IV	148	1.52 (0.99–2.34)	0.052

* *P*<0.05.

**Table 3:** Correlation of IKKs gene expression with OS in gastric cancer patients with HER2 expression status

IKKs	HER2 status	Cases	Low	High	HR (95% CI)	*P*-value
IKKα	Negative	532	270	262	0.50 (0.4–0.64)	3.7e-09*
	Positive	344	255	89	0.63 (0.46–0.86)	0.0033*
IKKβ	Negative	532	267	265	1.8 (1.43–2.26)	4e-07*
	Positive	344	85	259	1.75 (1.26–2.42)	7e-04*
IKKγ	Negative	532	199	333	2.29 (1.77–2.97)	1.1e-10*
	Positive	344	93	251	1.73 (1.27–2.35)	0.00045*
IKKε	Negative	532	378	154	1.61 (1.27–2.03)	6.8e-05*
	Positive	344	88	256	1.57 (1.15–2.15)	0.0041*
SIKE	Negative	532	393	139	1.80 (1.42–2.28)	1.1e-06*
	Positive	344	101	243	1.77 (1.3–2.42)	0.00025*

* *P*<0.05.

**Table 4:** Correlation of IKKs gene expression with OS in gastric cancer patients with different treatment strategy

IKKs	Treatment	Cases	HR (95% CI)	*P*-value
IKKα	Surgery alone	380	0.70 (0.53–0.94)	0.016*
	5 FU-based adjuvant	153	1.38 (0.93–2.04)	0.11
	Other adjuvant	76	0.25 (0.10–0.65)	0.0021*
IKKβ	Surgery alone	380	1.44 (1.07–1.92)	0.014*
	5 FU-based adjuvant	153	0.77 (0.54–1.11)	0.17
	Other adjuvant	76	0.46 (0.19–1.13)	0.084
IKKγ	Surgery alone	380	1.58 (1.11–2.26)	0.011*
	5 FU-based adjuvant	153	1.51 (1.04–2.2)	0.03*
	Other adjuvant	76	1.77 (0.70–4.44)	0.22
IKKε	Surgery alone	380	1.17 (0.88–1.56)	0.29
	5 FU-based adjuvant	153	1.84 (1.21–2.78)	0.0036*
	Other adjuvant	76	0.53 (0.21–1.33)	0.17
SIKE	Surgery alone	380	1.32 (0.99–1.77)	0.059
	5 FU-based adjuvant	153	1.90 (1.33–2.71)	3e-04*
	Other adjuvant	76	0.12 (0.02–0.92)	0.015*

* *P*<0.05.

**Table 5:** Correlation of IKKs gene expression with OS in gastric cancer patients with gender expression status

IKKs	Gender	Cases	HR (95% CI)	*P*-value
IKKα	Male	545	0.45 (0.35–0.57)	2.1e-11*
	Female	236	0.46 (0.32–0.67)	3.9e-05*
IKKβ	Male	545	2.10 (1.67–2.64)	9.7e-11*
	Female	236	1.88 (1.30–2.71)	0.00064*
IKKγ	Male	545	2.90 (2.21–3.82)	1.6e-15*
	Female	236	2.05 (1.44–2.92)	4.8e-05*
IKKε	Male	545	1.75 (1.42–2.17)	1.8e-07*
	Female	236	1.81 (1.26–2.6)	0.001*
SIKE	Male	545	1.63 (1.31–2.02)	7.9e-06*
	Female	236	2.38 (1.66–3.42)	1.4e-06*

* *P*<0.05.

**Table 6:** Correlation of IKKs gene expression with OS in gastric cancer patients with differentiation degree

IKKs	Treatment	Cases	HR (95% CI)	*P*-value
IKKα	Poor	165	1.36 (0.91–2.02)	0.13
	Moderate	67	0.57 (0.27–1.22)	0.14
	Good	32	0.60 (0.25–1.43)	0.24
IKKβ	Poor	165	0.81 (0.54–1.22)	0.31
	Moderate	67	2.06 (0.97–4.38)	0.055
	Good	32	2.71 (0.79–9.24)	0.097
IKKγ	Poor	165	0.75 (0.50–1.12)	0.16
	Moderate	67	1.69 (0.77–3.72)	0.18
	Good	32	4.84 (1.12–20.96)	0.02*
IKKε	Poor	165	0.81 (0.54–1.22)	0.31
	Moderate	67	2.06 (0.97–4.38)	0.055
	Good	32	2.71 (0.79–9.24)	0.097
SIKE	Poor	165	1.43 (0.94–2.17)	0.09
	Moderate	67	1.85 (0.96–3.58)	0.064
	Good	32	4.13 (0.96–17.82)	0.039*

* *P*<0.05.

The correlation of IKKs gene expression with OS in patients with known HER2 mutation was outlined in Table 3. IKKα showed better OS irrespective of HER2 status, with HER2 negative patients having a slightly lower HR = 0.5 (0.4–0.64), *P*=3.7e-09, when compared with HER2 positive patients 0.63 (0.46–0.86), *P*=0.0033, whereas all other IKKs subgroups showed worse OS, with HER2 negative status always having the higher HR of the two groups within the IKKs subgroup.

Table 4 showed a significant difference in IKKα expression and OS in the patients with the treatments of surgery alone and ‘other adjuvant’ therapy. The other adjuvant therapy group showed lower HR = 0.25 (0.1–0.65), *P*=0.0021 when compared with the surgical only group HR = 0.7 (0.53–0.94), *P*=0.016. IKKβ overexpression only showed significant difference in the surgical only group showing a worse OS with HR = 1.44 (1.07–1.92), *P*=0.014. In elevated IKKγ expression only surgical and 5 FU adjuvant groups (5 FU) showed significant difference with worse OS, surgical alone having the slightly higher HR = 1.58 (1.11–2.26), *P*=0.011, with 5 FU group HR = 1.51 (1.04–2.2), *P*=0.03. In the IKKε overexpression subgroup, only 5 FU showed a significant correlation, HR = 1.84 (1.21–2.78), *P*=0.0036. SIKE overexpression revealed significant correlation in both 5 FU and other adjuvant groups, with a worse OS in 5 FU group (HR = 1.9, 95% CI: 1.33–2.71, *P*=3e-04), however a better OS in the other adjuvant group (HR = 0.12, 95% CI: 0.02–0.92, *P*=0.015).

IKKα overexpression showed the better OS in both males and females however all following IKKs subgroups showed worse OS in both males and females (Table 5). In IKKβ and IKKγ overexpression, male gender conferred a higher HR whereas in IKKε and SIKE overexpression females showed the higher HR.

Degree of differentiation largely did not show significant correlation to OS as seen in Table 6, with the exception of IKKγ overexpression in good differentiation, with a poor OS (HR = 4.84, 95% CI: 1.12–20.96, *P*=0.02) and SIKE overexpression showing a similarly poor OS in the good differentiation group with HR = 4.13 (0.96–17.82), *P*=0.039.

## Discussion

The intent of our study was to investigate the prognostic value of IKKs and SIKE expression in patients with gastric cancer using the KM plotter, which, to our knowledge, had not been attempted by any previous study thus far.

Overexpression of IKKα was found to be positively correlated with OS rates in all gastric cancer patients, apart from mixed histological type. Additionally, the findings for IKKα held true at different developmental stages of gastric cancer, gender, as well as HER2 status. Conversely, overexpression of IKKβ, IKKγ, IKKε, and SIKE was associated with negative OS rates in all gastric cancer patients, except for mixed histological type in the latter isoforms. IKKβ was also found to decrease OS rates at all developmental stages of cancer.

Previous authors have suggested a tumor suppressor role for IKKα in squamous cell carcinomas of the skin, oral cavity, lung, and nasopharynx, based on the observation that, in many such cases, IKKα is either mutated or significantly down-regulated [12]. On the other hand, an oncogenic role for IKKα has also been proposed in cancers of the breast and prostate [12–14]. While our findings seem to indicate that IKKα falls into the former category of tumor suppressor in gastric cancer, further investigation is needed to identify the specific conditions responsible for eliciting these contrasting functions of IKKα in different cancer types.

The findings for SIKE are somewhat inconsistent with current understanding of its function as a suppressor of IKKε, the overexpression of which has been previously documented in ovarian and breast cancers [7]. In another study, IKKε was positively identified in 13.6% of gastric cancer patients, which suggests it may have some tumorigenic potential [7]. Other trials have found elevated levels of IKKε expression in cancers of the breast, ovaries, esophagus, and prostate [7,15–18] The data collected for IKKε appear to lend further credibility to these claims.

Many earlier studies have noted the role of IKKβ in oncogenesis. In breast cancer, IKKβ is thought to inhibit the pro-apoptotic transcription factor forkhead box protein O3a (FOXO3a), thereby inducing tumorigenesis [19]. Additionally, the overexpression of IKKβ has been linked to the metastatic progression of hepatocellular carcinoma, through the expression of factors such as receptor activator for NF-κB ligand (RANKL) and osteoprotegerin (OPG) [20]. Furthermore, higher expression of IKKβ in ovarian cancer samples is negatively correlated with OS [21]. Our own findings appear to be consistent with those of previous authors. To date, targetted therapy has tended to focus on selective inhibition of IKKβ as a means to inhibit the NF-κB pathway, but these have potential complications due to their importance in homeostatic functions [6].

The comparisons made on basis of HER2 status seemed to indicate that on average, HER2 negative status had worse OS rates compared with HER2 positive status, for all isoforms except for IKKα. Known primarily for its oncogenic role in breast cancer, overexpression of HER2 has also been found in ∼20% of gastric cancers, as well as cancers of the colon, bladder, endometrium, ovaries, and head and neck, amongst others [7,22]. *H. pylori* is thought to play a role in the overexpression of HER2 according to some trials [23]. Despite the success of trastuzumab as an HER2 inhibitor, our research indicates that further research is needed into targetted therapeutic agents in gastric cancer.

Statistical differences were also observed when comparing the subjects on the basis of treatment modalities. As remarked earlier, IKKα was the only subject associated with better OS rates, whereas all other isoforms were associated with poorer OS rates. Those isoforms associated with poorer OS rates may be potential targets for therapeutic research in the future. When compared on the basis of gender, only IKKα demonstrated better survival rates, whereas the other isoforms consistently demonstrated worse survival rates. Except for IKKβ and IKKγ, the remaining isoforms demonstrated better survival rates for women compared with men. Finally, comparison based on degree of differentiation was found to be mostly insignificant.

Based on our results, it is evident that further research is required to elucidate the effects of therapeutic modalities, histopathological type, and HER2 status on IKK and SIKE expression in gastric cancer. While trastuzumab and ramucirumab have been successful, there remains a need for more therapeutic options to improve outcomes for patients. To that end, IKKβ, IKKγ, IKKε, and SIKE show potential as therapeutic targets based on our findings and should be the focus of investigation in the future.

## Conclusion

Using the comprehensive survival analysis platforms of KM plotter as seen above, our results show that of the five members analyzing mRNA, only expression of IKKα was significantly correlated with favorable OS in gastric cancer. Conversely mRNA expression of IKKβ, IKKγ, IKKε, and SIKE were all generally associated with unfavorable OS in gastric cancer. In addition, these members were observed to continue playing an important function as predictors of OS across pathological stages, HER2 expression status, treatment strategies, and genders. Remarkably IKKε showed better OS in stage IV gastric cancer, also of note both IKKε and SIKE expression were linked to better OS in diffuse type gastric cancer. The results indicate a strong prognostic significance amongst IKKs and SIKE which may be utilized in assessing OS. Despite our findings being statistically significant, the mechanism for these seemingly paradoxical results remain unclear and merits further research. Furthermore, we recommend further research into the use of IKKs and SIKE as possible therapeutic targets in gastric cancer.

## Abbreviations

CI: confidence interval
HER2: human epidermal growth factor receptor 2
HR: hazard ratio
IκB: inhibitor of κB
IKK: inhibitory κB kinase
IKKα: inhibitor of nuclear factor κB kinase subunit α
IKKβ: inhibitor of nuclear factor κB kinase subunit β
IKKγ(NEMO): inhibitor of nuclear factor κB kinase subunit γ
IKKε: inhibitor of nuclear factor κB kinase subunit ε
KM plotter: Kaplan–Meier plotter
NF-κB: nuclear factor κB
OS: overall survival
SIKE: suppressor of IKKε
TBK1: TANK-binding kinase 1
