# Expression of Concern: Prognostic values of inhibitory κB kinases mRNA expression in human gastric cancer

**DOI:** 10.1042/BSR-20180617_EOC

**Published:** 2021-04-06

**Keywords:** gastric cancer, Gene Expression Omnibus, Inhibitory κB Kinases, nuclear factor κB, prognosis

The Editorial Office has been made aware of potential issues surrounding the scientific validity of this paper, hence has issued an expression of concern to notify readers whilst the Editorial Office investigates.

