# Correction: Prognostic values of inhibitory κB kinases mRNA expression in human gastric cancer

**DOI:** 10.1042/BSR-2018-0617_COR

**Published:** 2021-07-01

**Keywords:** gastric cancer, Inhibitory kappa B Kinases, nuclear factor kappa B, prognosis

This Correction follows an Expression of Concern relating to this article previously published by Portland Press.

The Authors of the original article “Prognostic values of inhibitory κB kinases mRNA expression in human gastric cancer” (*Biosci Rep* (2019) 39(1); DOI: 10.1042/BSR20180617) would like to correct their Figure 2 graphs.

**Figure 2:**
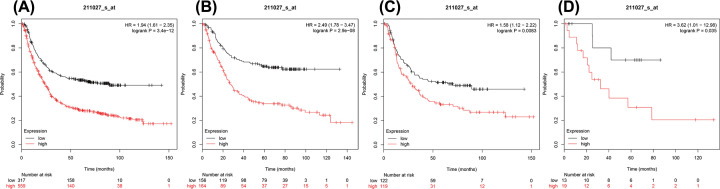
The prognostic value of IKKβ expression in gastric cancer (**A**) All the patients; (**B**) intestinal cancer patients; (**C**) diffuse cancer patients; and (**D**) mixed cancer patients.

The Authors state that due to an error in the initial drafting process of the article, a graph was accidentally duplicated in parts B and C of Figure 2. This duplication had not been identified at an earlier stage, and was therefore carried through to the publication. The correct Figure 2 graphs are presented here. The Authors declare that this error has no effect on the conclusions of their study.

